# An *in vitro* evaluation of biofilm removal from simulated root canals using sodium hypochlorite irrigation solution at various temperature settings

**DOI:** 10.1371/journal.pone.0325431

**Published:** 2025-06-23

**Authors:** Kevser Şenel, Ismail Uzun, Rawan Alqawasmi

**Affiliations:** 1 Endodontist, Ordu Central Dental Hospital, Ordu, Turkey; 2 Department of Endodontics, Faculty of Dentistry, Ondokuz Mayis University, Samsun, Turkey; 3 Department of Endodontics, Faculty of Dentistry, Arab American University, Jenin, Palestine; University of Puthisastra, CAMBODIA

## Abstract

**Background:**

Using sodium hypochlorite (NaOCl) irrigation solution at various temperatures is common for removing biofilms in root canals and the isthmus. Numerous studies have examined the impact of temperature on biofilm removal in extracted teeth. However, this study aimed to assess the effect of needle irrigation using NaOCl solution heated to different temperatures on the structure of *E. faecalis* biofilm in artificial teeth produced by 3D printing technology.

**Materials and methods:**

The isthmus in the mesial canals of 55 artificial tooth samples, which were produced from the 3D model obtained by micro-CT of the mandibular first molars, was evaluated. The standard strain *E. faecalis* ATCC 19433 was used to infect artificial tooth specimens. The samples were divided into a control group and four experimental groups receiving sodium hypochlorite solutions at 21°C, 45°C, 60°C, and 150°C. Following irrigation, scanning electron microscope (SEM) imaging was conducted at varying magnifications to visualize the remaining biofilm areas in the root canals and the isthmus. The ImageJ program quantified biofilm areas in the isthmus region. Statistical analyses, including Shapiro-Wilks, Kruskal Wallis H, and t-tests, were conducted on the measurements. A p-value of < 0.05 was considered statistically significant.

**Conclusions:**

The results did not differ between the control and 21 °C groups (p > 0.05). However, removal areas were larger in the 45°C, 60°C, and 150°C groups than in the control group (p < 0.05). No difference was observed in the biofilm removal efficiency in different isthmus regions (p > 0.05). The findings revealed that an increase in temperature enlarged the removal areas.

## Introduction

The success of endodontic treatment depends on eliminating microorganisms from the root canal system and preventing reinfection [1]. Microorganisms often persist in anatomical structures such as the isthmus, accessory canals, and apical ramifications These structures are difficult to clean using traditional methods, leading to recurrent infections and lower treatment success rates [2]. To improve treatment success, it is important to enhance the mechanical washing effect and chemical capacity of irrigation solutions to disinfect the root canal system, as microorganisms cannot be removed entirely through chemical preparation alone [3].

Sodium hypochlorite (NaOCl) is commonly used in endodontics because it dissolves tissues and kills microbes [4]. Its effectiveness can be improved by increasing concentration and volume, heating, lowering pH, and using agitation methods [5]. However, as the concentration increases, so does cytotoxicity [6]. Using heat-activated low-concentration NaOCl can reduce cytotoxicity while maintaining effectiveness [7]. Increasing the temperature or using surfactants can also improve solution penetration and increase antibacterial activity.

Heating sodium hypochlorite has been shown to enhance its physicochemical properties and antimicrobial performance. Elevated temperatures reduce the surface tension of NaOCl, allowing it to penetrate more effectively into dentinal tubules and complex anatomical structures, such as the isthmus and lateral canals [3]. Moreover, increased temperature accelerates the decomposition of NaOCl into hypochlorous acid and other reactive species, thereby improving its tissue dissolution capacity and antibacterial activity [8,9]. Studies have also reported that intracanal heating maintains the temperature of the solution for longer periods compared to pre-heating methods, enhancing its efficacy within the root canal system. These findings have led to increased interest in using heated NaOCl as an adjunctive disinfection strategy in endodontics.

The root canal system presents a complex internal morphology that includes anatomical variations such as isthmuses, apical ramifications, accessory canals, and lateral canals. These structures can harbor residual microorganisms even after conventional instrumentation and irrigation, complicating disinfection efforts and increasing the risk of treatment failure [2,10,11]. In particular, the presence of isthmuses—narrow connections between root canals—poses significant challenges for effective irrigation due to their limited accessibility. Understanding the influence of anatomical intricacy on irrigation efficacy is essential for optimizing endodontic disinfection protocols.

The root canal system is typically colonized by a complex polymicrobial community dominated by facultative and obligate anaerobes, including *Enterococcus faecalis*, *Fusobacterium*, *Prevotella*, and *Porphyromonas* species. These microorganisms often form organized biofilms within the canal space and dentinal tubules, enhancing their resistance to antimicrobial agents and host immune responses [12]. Among them, *E. faecalis* is particularly persistent due to its ability to survive under nutrient-deprived conditions and penetrate deeply into dentinal tubules [13]. Sodium hypochlorite (NaOCl) is widely used in endodontics because of its potent tissue-dissolving and broad-spectrum antimicrobial properties, which are critical for disrupting biofilms and eradicating resistant bacterial populations within the root canal system [14].

3D printing is a rapid prototyping technique using 3D printers to create models of specialized materials based on designs made with computer-aided engineering (CAE) and computer-aided design (CAD) software. This technology facilitates the quick and precise production of highly complex materials custom-made for the patient or replicas needed in large numbers for scientific research [15]. In recent years, 3D printing has become the primary production technology in several fields of healthcare and medicine, including dentistry, tissue engineering and regenerative medicine, medical devices, anatomical models for use in surgical planning or education, and drug formulations [16]. In dentistry, 3D printing offers a promising way to quickly produce high-resolution natural tooth replicas. This approach allows for the standardization of samples in (in vitro) studies that examine treatments such as root canal instrumentation, filling, and retreatment.

Although several studies have investigated the antibacterial effectiveness of NaOCl at different temperatures in extracted teeth [17], significant anatomical variability limits the reproducibility and standardization of such experiments [18]. Furthermore, the specific impact of heated NaOCl on biofilm removal within isthmus structures remains insufficiently explored in controlled models [19]. This study aims to address this gap by utilizing 3D-printed artificial molars exhibiting a standardized isthmus morphology. Such a technique, would allow for a controlled assessment of biofilm removal efficiency under varying thermal conditions.

This study used anatomically standardized 3D-printed root canal models derived from the mesial root of mandibular molars, replicating Type V isthmus morphology as defined by Hsu and Kim [20]. The 3D-printed models allows for consistent sample standardization not offered by natural teeth. Additionally, intracanal heating of NaOCl to 150°C using a heat carrier—tested in this study—has not been previously assessed in such a model. This offers a novel perspective on the efficacy of heated NaOCl irrigation in complex root anatomy under controlled conditions.

Using in vitro biofilm models in microbiological studies offers several advantages, such as ease of modification when necessary, variable control, low cost, and ease of replication. Additionally, it provides the preliminary data needed for future validation through in vivo testing [21]. This study aims to investigate the biofilm removal efficiency of isthmus structures formed in an artificial root canal model by irrigating with NaOCl heated to various temperatures using a needle. The null hypothesis of this study was that there would be no significant difference in the amount of biofilm removed.

## Materials and methods

### Ethical approval

Since the single tooth used in this study was extracted for routine clinical purposes unrelated to the research and was collected anonymously, informed consent from the participant was not required. This study was approved by the Clinical Research Ethics Committee of Ondokuz Mayis University (Approval No: 2022/369). The manuscript of this laboratory study has been written according to the Preferred Reporting Items for Laboratory Studies in Endodontology (PRILE) 2021 guidelines.

**Fig 1:**
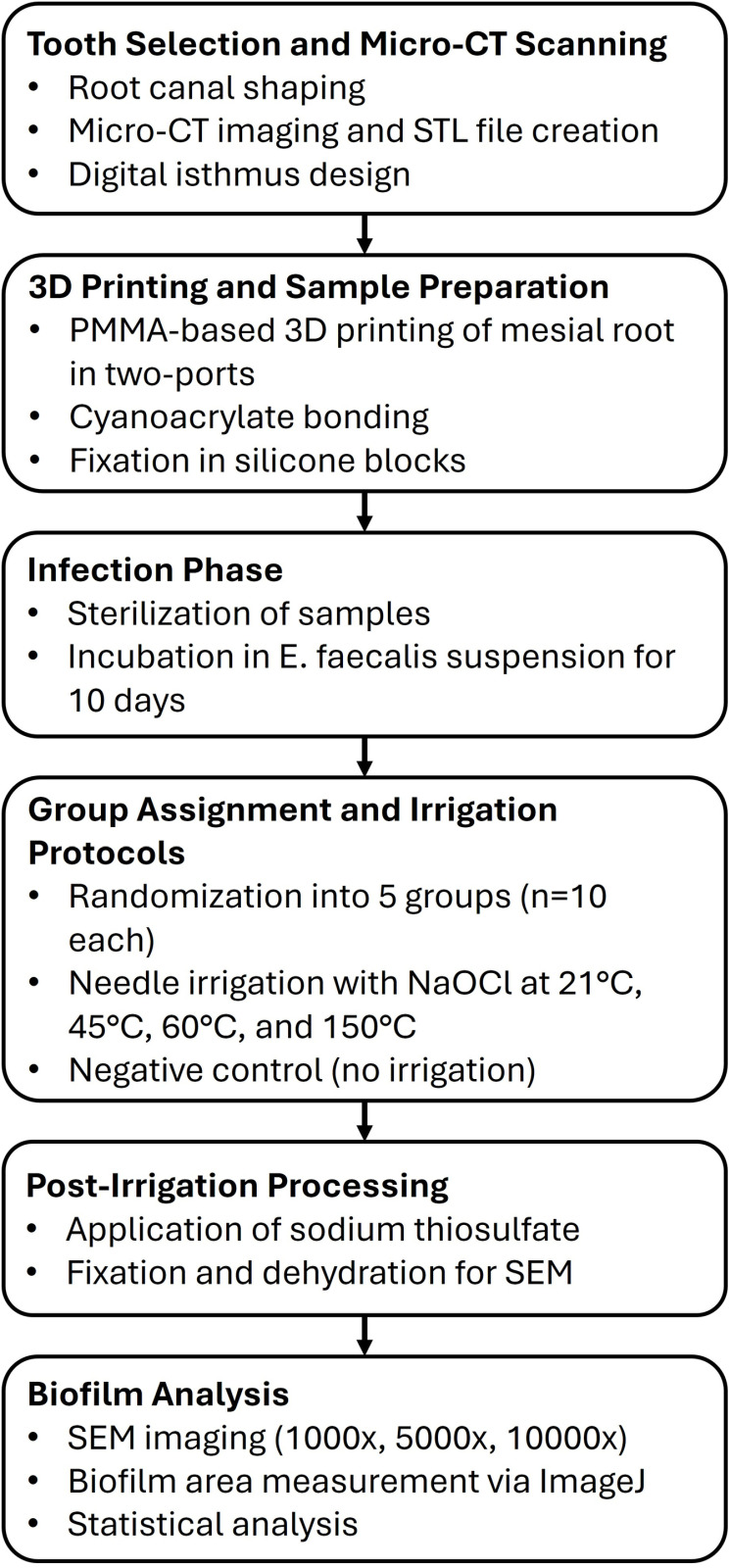
Workflow of the experimental protocol, including 3D model generation, irrigation procedures, and SEM analysis steps.

An overview of the experimental workflow is presented in Fig 1.

### Sample size calculation

A power analysis was performed prior to the study to determine the minimum sample size required. Sample size was determined using G*Power 3.1 software (Heinrich Heine University Düsseldorf, Düsseldorf, Germany). Based on the study by Mohmmed SA et al., titled *“Investigation into in situ Enterococcus faecalis biofilm removal by passive and active sodium hypochlorite irrigation delivered into the lateral canal of a simulated root canal model”*, the effect size (Cohen’s f) was determined to be 0.464. Assuming an alpha value of 0.05 and a statistical power of 0.80, the minimum required sample size was calculated to be 10 specimens per group. Accordingly, 55 artificial tooth samples were included in the study.

### Sample preparation

Fig 1. PRILE 2021-compliant flowchart illustrating the experimental workflow including sample preparation, group allocation, treatment procedures, and analysis. The study selected a mandibular first molar with mesial root configuration Vertucci type II root canal that had no caries, restoration, or crown root fracture. Root canal shaping of the mandibular first molar was performed using an electric endodontic motor (VDW.GOLD; VDW Dental, Munich, Germany) and nickel-titanium files (ProTaper Next; Dentsply Maillefer, Ballaigues, Switzerland) following the opening of the endodontic access cavity, utilizing the ‘crown-down’ technique. The final shaping of the mesial root canals was completed with X2 (#25.06) and the distal root canal with X3 (#30.07) canal files. At each file change, irrigation was performed with 2 ml of 2.5% NaOCl for each canal. After the root canal shaping process was completed, in the final wash protocol, the root canals were washed with 2 ml of ethylenediaminetetraacetic acid (EDTA), distilled water, and NaOCl. The root canal was than rewashed with 2 ml of distilled water to eliminate the effect of irrigation solutionsand dried with paper cones.

### Micro-CT scanning and model preparation

**Fig 2:**
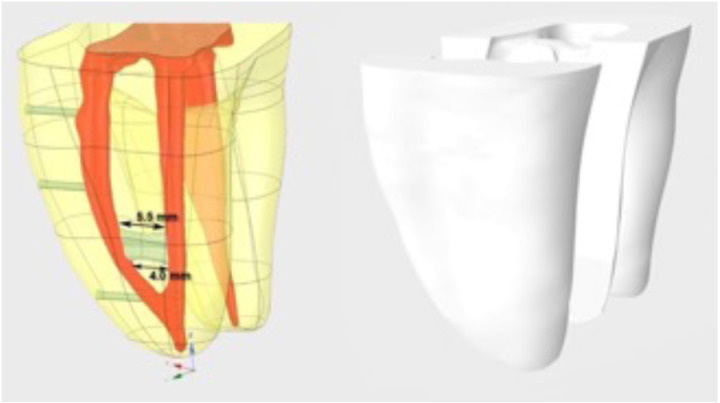
Micro-CT–based 3D reconstruction of the mandibular molar model showing the simulated isthmus in the mesial root.

After root canal shaping was completed, the sample was scanned using a SkyScan micro CT (SkyScan 1172 X-ray microtomography, Antwerp, Belgium) device. At the end of the scan, 2275 raw images were obtained and saved in. stl format with CTAn software (Bruker, Belgium). In the recorded images, a 3D root canal model with a working length of 13.2 mm was obtained by cutting the crown at the level of the enamel-cementum border. In the mesial root of this model, a structure resembling a Type V isthmus (based on Hsu and Kim’s isthmus classification) [20] commonly found in the Turkish population, was formed approximately 45.5 mm coronally from the mesiobuccal and mesiolingual canals (Fig 2) [22].

The mesial root of the 3D model was virtually divided into two symmetrical halves in the vertical plane to facilitate post-procedural internal analysis. Following the power analysis conducted to ascertain the requisite sample size for the study, a minimum of 10 samples per group was necessary to ensure statistical validity. The 3D tooth model was transferred to the EnvisionTEC Vida 3D dental printer (Ultra® 3SP™ Family, ve Prefactory® Family) in STL format, and 55 replica teeth were obtained using polymethyl methacrylate (PMMA) acrylic printing material.

Each half of the mesial root was printed separately, and the two parts were subsequently bonded using cyanoacrylate adhesive to create a unified artificial tooth specimen. These specimens were embedded in silicone blocks to ensure stability during the irrigation procedures. Artificial tooth samples were then infected using the standard strain *E. faecalis* ATCC 19433 to allow biofilm formation. After completion of the irrigation and neutralization protocols, each sample was carefully re-separated along the original bonding line using a sterile surgical scalpel. This method enabled direct visualization of the internal canal structure and isthmus region during SEM imaging without disrupting the remaining biofilm layer.

### Infection procedure

A suspension was prepared with TSB medium using the E. faecalis ATCC 19433 standard strain. From this suspension, 4 ml of each was transferred to centrifuge tubes. The replicate tooth samples were sterilized in a steam autoclave at 121°C under 15 psi pressure for 15 minutes and then incubated in the prepared suspension at 37°C for 10 days. This prosedure followed previously established protocols to allow the development of mature and stable *E. faecalis* biofilms suitable for disinfection studies [23,24]. The medium was refreshed during incubation on days three and six. After incubation, the medium was aspirated, and the replicate tooth specimens were gently washed with saline.

### Experimental procedures

**Fig 3:**
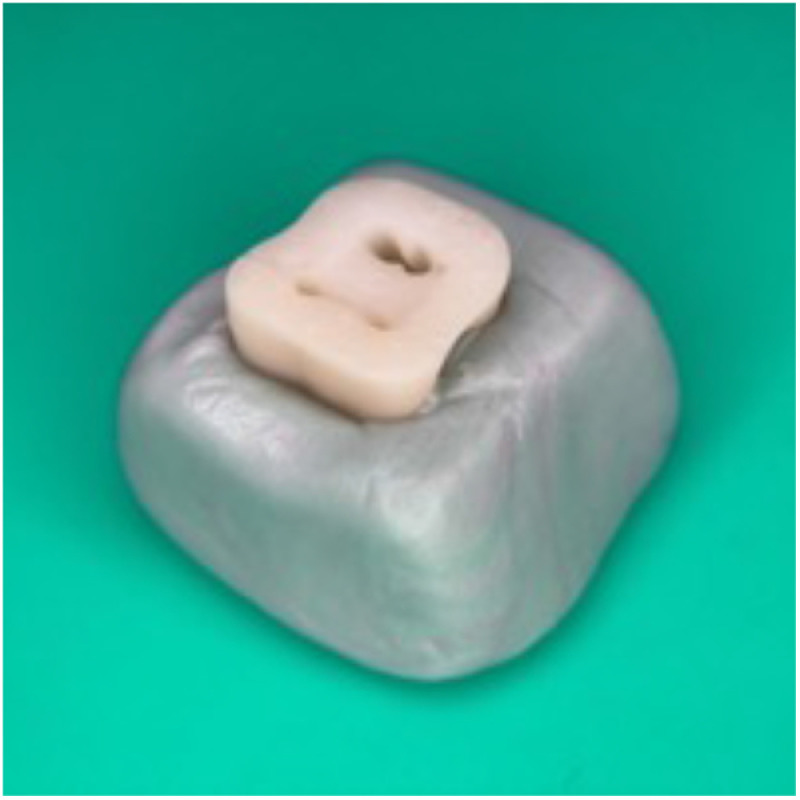
Photograph of a 3D-printed artificial tooth model fixed into a silicone block before irrigation.

After biofilm formation, all specimens were randomly assigned to experimental groups using a computer-generated random number table. The allocation was performed by an investigator blinded to the group assignments to minimize selection bias. Five of the 55 replica samples used in our study were randomly selected as a negative control group. The remaining 50 replicates were randomly divided into five experimental groups (n = 10), with ten samples in each group. The root surface of all specimens was covered with cyanoacrylate, and the tooth models were fixed on silicone blocks (Fig 3).

All experiments were conducted in an oven at 37°C to mimic body temperature. Temperatures of 21°C, 45°C, 60°C, and 150°C were selected based on previous studies and clinical protocols. While 21°C and 45°C represent commonly used pre-heating strategies, 60°C has been associated with improved chemical efficacy without significant vaporization. The 150°C temperature corresponds to the tip temperature of intracanal heating devices such as System B or Fi-P heat carriers, which are commonly used in contemporary endodontic practice. The groups were as follows:

- The biofilm control group (**Group 1**) did not receive any irrigation.
- In the 21°C NaOCl group (**Group 2**), the mesial root canals of the samples infected with *E. faecalis* were irrigated at 21°C with 2.5% NaOCl using a 30-gauge side-vented NaviTip irrigation needle (Ultradent Products Inc., South Jordan, UT, USA), 2 mm shorter than the working length. The needle was moved 3–5 mm up and down during irrigation to prevent getting stuck in the canal. Irrigation was applied to the mesiobuccal and mesiolingual canals for 120 seconds using 6 ml of NaOCl for each root canal.
- In the 45°C NaOCl group (**Group 3**), disposable syringes containing 2 ml sodium hypochlorite solution were placed in a 45°C water bath for 30 minutes before irrigation. The mesial root canals of specimens infected with *E. faecalis* biofilm were irrigated using the same procedures with NaOCl heated to 45°C.
- In the 60°C NaOCl group (**Group 4),** the root canals of the specimens infected with *E. faecalis* biofilm were irrigated by applying the same protocols with NaOCl heated in a 60°C water bath.
- In the 150°C NaOCl group (**Group 5**), the root canals of the samples infected with *E. faecalis* biofilm were first irrigated with 2 ml of 2.5% NaOCl at room temperature of 21°C. Then, an endodontic heat source (Fi-P, Guilin Woodpecker Medical Instrument Co., Ltd., Guilin, China) with a temperature of 150°C was inserted into the canal 2 mm shorter than the working length using a 35/04 diameter cap. The device was activated for 10 seconds. During the heating process, the heat carrier was moved back and forth in the canal to prevent it from getting stuck. The heat source was removed from the canal, and after 20 seconds, the NaOCl solution was refreshed. This procedure was repeated three times.

**Fig 4:**
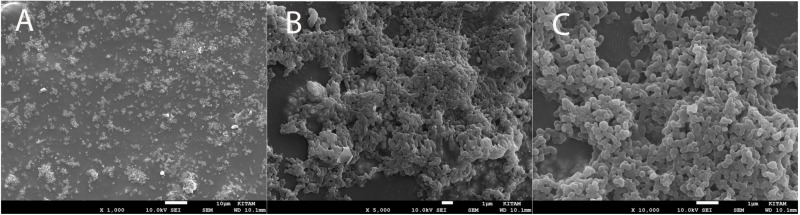
SEM images of the isthmus buccal region captured at 1000 × , 5000 × , and 10000 × magnifications, demonstrating progressive visualization of biofilm structure. Images are from a representative specimen for illustrative purposes.

After the irrigation procedures in all groups, each root canal was washed with 2 ml of 10% sodium thiosulfate to terminate the effect of the NaOCl irrigation solution. After the irrigation procedures, all samples were removed from the silicone blocks, divided into two parts again with the help of a surgical scalpel and prepared for SEM imaging. For this, replica samples were kept in 4% glutaraldehyde for 1 hour at room temperature. Then, the samples were kept in 70%, 80%, 90%, and 96% pure ethyl alcohol (Isolab, Bavyera, Germany) at room temperature for 10 minutes each. After the final alcohol solution was aspirated, the samples were left to dry in a dark environment at room temperature. Prior to imaging, the samples were coated with a thin conductive layer of gold-palladium (Au/Pd) using a sputter coater (Quorum Q150R Plus, Quorum Technologies, UK). The coating was applied under a vacuum of 0.1 mbar with a current of 20 mA for 60 seconds, resulting in an approximate coating thickness of 10 nm, as recommended for high-resolution biological samples. Imaging was performed at Ondokuz Mayis University, Black Sea Advanced Technology Research and Application Center (OMÜ-KİTAM) using a n SEM (SEM; JSM-7001F, JEOL Ltd., Tokyo, Japan). The biofilm formation in each sample was confirmed with imaging under magnification with the SEM, images of the regions were then taken. The isthmus region of each sample was divided into three regions: buccal, middle, and lingual.Pictures were taken with the SEM at 1,000x, 5,000x, and 10,000x magnifications, and then recorded (Fig 4).

### Image analysis

Biofilm areas in the recorded images were measured using the ImageJ image analysis program (ImageJ software, version 1.53t; National Institutes of Health, Bethesda, MD, USA). SEM images were initially captured at 10,000x magnification to confirm the presence of biofilm in the buccal, middle, and lingual regions of the isthmus. Following this confirmation, images at 1,000x magnification were used for the quantitative analysis of the biofilm-covered areas. For this purpose, SEM images in JPEG format were imported into the software, and the scale was calibrated using the scale bar embedded in each SEM image. Biofilm remnants were identified using the thresholding tool, which highlighted the target areas, the software automatically calculated the surface area of the biofilm. This procedure was repeated for all three regions (buccal, middle, and lingual) of each specimen.

### Statistical analysis

The data obtained in this study were statistically evaluated using the RStudio (RStudio Team, 2022, Boston, MA, USA) program. Quantitative data were summarized as mean and standard deviation, and qualitative data were summarized as numbers. The conformity of the data to the normal distribution according to the groups was evaluated using the Shapiro-Wilk test, which determined that the values were not suitable for normal distribution (p < 0.001). Therefore, the Kruskal-Wallis H test was used to compare the groups’ mean areas of biofilms. Dunn’s test was used as the grouping method in non-parametric analyses, utilizing the R Package FSA program. Biofilm areas measured in different regions of the root canals are normally distributed. A t-test was used to compare these regions. The result was a p value of < 0.05 was considered statistically significant.

## Results

**Fig 5:**
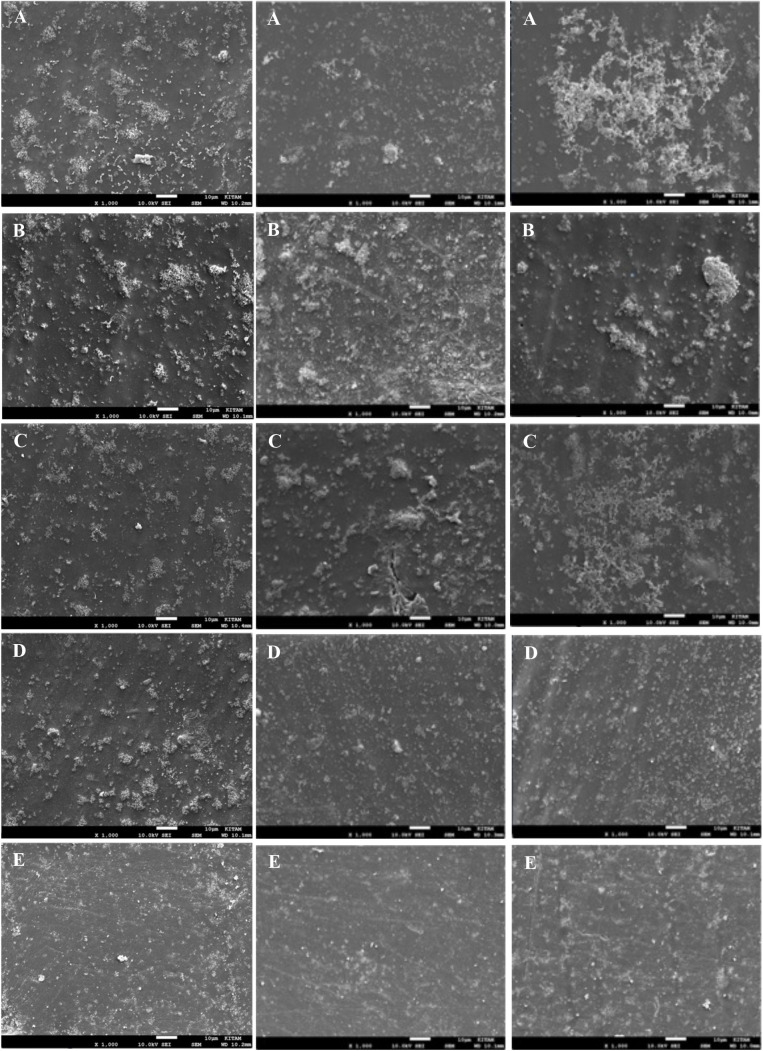
Representative SEM images of the biofilm structure observed in the buccal (left column), middle (center column), and lingual (right column) regions of the isthmus at 1000 × magnification. (A) Biofilm control group, (B) 21°C NaOCl group, (C) 45°C NaOCl group, (D) 60°C NaOCl group, and (E) 150°C NaOCl group. Increased biofilm clearance is visually observed as the irrigation temperature increases from A to E.

**Fig 6:**
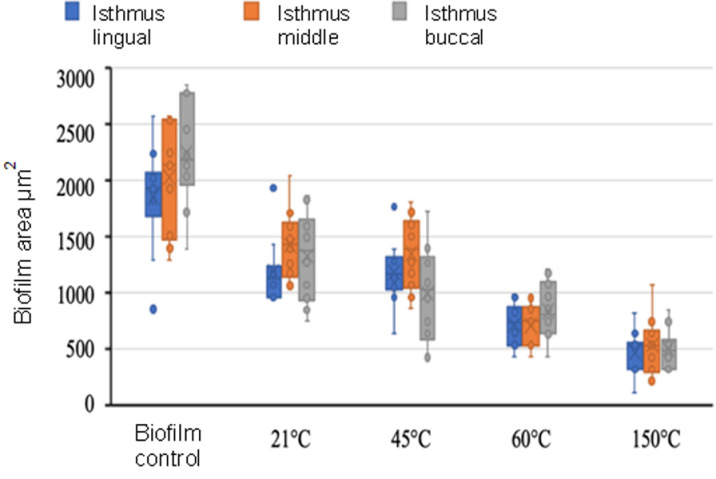
Boxplot comparison of biofilm area measurements in buccal, middle, and lingual isthmus regions across different temperature groups. “x” indicates the mean; the horizontal line indicates the median. Whiskers represent the 1st and 4th quartiles.

This study investigated microbiological changes in root canals by applying sodium hypochlorite (NaOCl) irrigation at different temperatures to artificial root canals infected with E. faecalis under laboratory conditions. Biofilm areas were quantified from the SEM images using ImageJ software. Bacterial growth in the biofilm control group was confirmed by SEM imaging (Fig 5). A quantitative comparison of biofilm areas across the different temperature groups demonstrated a progressive reduction in biofilm with increasing irrigation temperature, as shown in the boxplot analysis (Fig 6).

The biofilm areas in the buccal, middle, and lingual regions of the isthmus were evaluated based on the results of the comparative statistical data within and between groups. In all groups, there was no statistically significant difference between the regions in the same experimental group related to the biofilm areas remaining in the buccal, middle, and lingual regions of the isthmus (p > 0.05). The details are given in Table 2 (Fig 5). However, the mean areas among the groups differed, except for the 21ºC and 45ºC groups. The average of total biofilm areas for the isthmus in control, 21ºC, 45ºC, 60ºC, and 150ºC were 20,39 x10^-4^ mm^2^, 12,17 x10^-4^ mm^2^, 11,78 x10^-4^ mm^2^, 7,48 x10^-4^ mm^2^, and 5,34 x10^-4^ mm^2^, respectively. The mean and median values of each group are very close. The details of the findings are summarized in Table 1—the mean total biofilm areas in the isthmus decrease when the temperature increases.

**Table 1:** Biofilm areas in five experimental groups.

Groups	Minimum ± Maximum (x10^-4^mm^2^)	Median (x10^-4^ mm^2^)	Mean ± Standard deviation (x10^-4^mm^2^)
**Group 1 (Control)**	8.56 ± 28.47 ^a^	20.39	20.42 ± 4.92
**Group 2 (21°C)**	7.50 ± 20.41^b^	12.17	13.14 ± 3.43
**Group 3 (45°C)**	4.26 ± 18.13 ^b^	11.78	11.76 ± 3.74
**Group 4 (60°C)**	4.26 ± 12.16 ^c^	7.48	7.52 ± 2.22
**Group 5 (150°C)**	1.06 ± 10.69 ^d^	5.34	5.01 ± 2.12

**Note:** Data are presented as median values (x10 ⁻ ⁴ mm²), along with minimum–maximum and mean ± SD. Superscript letters indicate statistically significant differences between groups (Kruskal–Wallis test with post hoc pairwise comparisons, p < 0.05).

**Table 2:** Median biofilm areas in isthmus buccal, middle, and lingual regions.

Median (x10^-4^mm^2^)			
Groups	Isthmus buccal	Isthmus middle	Isthmus lingual
**Group 1 (Control)**	20.185 ^a^	20.141 ^a^	10.929 ^a^
**Group 2 (21°C)**	10.382 ^ab^	10.436 ^a^	10.121 ^ab^
**Group 3 (45°C)**	10.023 ^bc^	10.389 ^ab^	10.164 ^ab^
**Group 4 (60°C)**	8.016 ^cd^	7.465 ^bc^	7.161 ^bc^
**Group 5 (150°C)**	4.810 ^cd^	5.343 ^c^	5.330^c^

**Note:** Data are presented as median biofilm areas (x10 ⁻ ⁴ mm²). Superscript letters within each column denote statistically significant differences between groups (Kruskal-Wallis test, p < 0.05).

Our findings for the buccal region revealed that the median biofilm areas (x10^-4^) in the control, 21ºC, 45ºC, 60ºC, and 150ºC groups were 20,185 mm^2^, 10,382 mm^2^, 10.023 mm^2^, 8,016 mm^2^, and 54.810 mm^2^, respectively. The biofilm area of the 21ºC group did not differ from the areas of the control group (p > 0.05). The biofilm areas in the groups 45ºC, 60ºC, and 150ºC were smaller than that of the control group (p < 0.05). The results showed that the increase in temperature resulted in a decrease in the average biofilm areas in groups (Table 2).

The median biofilm areas (x10 ⁻ ⁴ mm²) in the middle and lingual regions of the isthmus followed a similar pattern across the groups. In the middle region, the median values were 20.141 (control), 10.436 (21°C), 10.389 (45°C), 7.465 (60°C), and 5.343 (150°C). In the lingual region, the corresponding values were 10.929, 10.121, 10.164, 7.161, and 5.330, respectively. No statistically significant differences were observed between the control, 21°C, and 45°C groups (p > 0.05). However, the biofilm areas in the 60°C and 150°C groups were significantly smaller compared to the control group (p < 0.05). These findings indicate that increasing the temperature of NaOCl enhances biofilm removal in both the middle and lingual regions of the isthmus, consistent with the results observed in the buccal region (Table 2).

## Discussion

The success of endodontic treatment relies on effectively cleaning the root canal system through chemical and mechanical debridement and disinfection methods. While rotary instruments are commonly used, they are limited in accessing anatomical variations such as accessory canals, isthmuses, and apical ramifications [25]. These areas can accumulate debris, microorganisms, and by-products, leading to persistent periapical infections and hindering the proper adaptation of canal-filling materials [22]. Therefore, the importance of irrigation in conjunction with root canal instrumentation must be balanced for effective root canal cleaning.

*E. faecalis* is a pathogenic microorganism responsible for various forms of periradicular disease, ranging from primary endodontic infections to persistent periradicular infections [26]. This facultative, anaerobic, gram-positive bacterium is often associated with recurrent infections and can form biofilms within dentinal tubules, rendering it highly resistant to antimicrobial agents [27]. The biofilm-forming ability of *E. faecalis* is its most significant virulence factor, making it 1000 times more resistant to antimicrobial agents than planktonic forms. Consequently, *E. faecalis* is frequently employed in endodontic studies.

NaOCl is the preferred irrigation solution in root canal treatment due to its bactericidal activity, lubricating properties, ability to dissolve organic compounds, and low surface tension [28]. Complete removal and destruction of bacteria within the biofilm structure requires direct contact with the NaOCl irrigation solution, as emphasized in Moorer and Wesselink [29]. However, mature and aggregated biofilms within root canals can penetrate deep into dentinal tubules, compromising the antibacterial efficacy of the solution. Consequently, various activation methods are employed to enhance the effectiveness of NaOCl irrigation [30].

While higher concentrations of NaOCl exhibit superior antibacterial activity against *E. faecalis*, increased concentration also escalates its toxic properties [31]. Studies have demonstrated that heating low-concentration NaOCl solutions can enhance their efficiency. In their research, Sirtes et al. utilized pre-warmed NaOCl solutions for root canal irrigation and reported increased tissue solvent/antimicrobial activity with low-concentration NaOCl solutions [3]. Cunningham and Joseph also demonstrated that a NaOCl solution at body temperature allowed for faster disinfection than at room temperature [9].

Studies evaluating the temperature change when a preheated NaOCl irrigation solution is used in the canal have shown heated NaOCI equilibration Allowing body temperature to be reached quickly [32]. This suggests that heating the NaOCl solution in the canal may be a more effective activation method. Iandolo et al. [8] compared different irrigation methods for removing debris and smear layer from root canals Their method included saline solution at room temperature, NaOCl heated to 50 °C outside the canal, and NaOCl heated to 180 °C in the canal using extracted single-rooted human teeth [8]. Their results demonstrated that heating NaOCl to 180 °C in the canal was more effective in removing debris and the smear layer.

Similarly, Yared and Ramli investigated the antibacterial activity of various irrigation techniques, such as standard needle irrigation, sonic and ultrasonic activation, and heated NaOCl in canals infected with *E. faecalis* using extracted human lower premolar teeth [19]. They found that heating NaOCl in the canal was more effective in killing bacteria than conventional needle irrigation and sonic/ultrasonic activation. Consistent with these findings, our study showed that heating NaOCl to 150 °C in the canal significantly outperformed standard needle irrigation in removing *E. faecalis* biofilm. However, our study differed from previous ones as we used artificial tooth samples for standardization and evaluated the biofilm removal efficiency of preheated NaOCl from the isthmus area outside the canal.

Additionally, Pereira et al. investigated root canal irrigation’s chemical and mechanical effects on biofilm removal in the isthmus and lateral canals of artificial root canals. They found that heated sodium hypochlorite applied at a low flow rate was inferior to needle irrigation method. Similarly, our study used needle irrigation at a low flow rate of 0.05 ml/s, consistent with Pereira et al. and found that heated sodium hypochlorite was more effective than room-temperature sodium hypochlorite in removing biofilm from the isthmus area [18].

In this study, mandibular first molars were selected as the model tooth due to their high prevalence of anatomical complexities, particularly in the mesial root. Several studies have reported that the incidence of isthmuses in the mesial roots of mandibular molars can be as high as 83%, making them a relevant model for evaluating irrigation efficacy [33,34]. Additionally, the prevalence of Type V isthmus morphology, as described by Hsu and Kim, is notably high in these teeth, especially in populations of certain geographic regions such as Turkey [20,22]. Therefore, the use of a 3D-printed mandibular molar with a simulated isthmus structure provided a realistic and standardized experimental model for investigating the effect of heated NaOCl on biofilm removal.

In studies involving extracted teeth, achieving standardization is challenging despite selecting samples based on specific criteria. Selected specimens can exhibit variations in apical ramifications, lateral canals, isthmuses, and root canal angles and curvatures. These anatomical differences can impact experimental outcomes. Some studies have utilized acrylic blocks or tooth models that simulate human teeth to address this standardization issue. Using these materials as substrates allows for better standardization as their size and structural properties are consistent [35]. Synthetic models created through 3D printing techniques have also been employed to obtain large numbers of samples with uniform anatomical characteristics and improved resolution [36].

However, it should be noted that these synthetic materials lack the unique organic-inorganic structure of dentin and dentinal tubules Furthermore, they may not fully replicate clinical conditions due to potential differences in microorganism adhesion compared to natural surfaces [35]. Nevertheless, studies investigating 3D printing materials have demonstrated that they enable the attachment and growth of *E. faecalis* biofilm to a similar extent as dentin surfaces [37]. Based the available literature, our study employed acrylic root canal models created through 3D printing using PMMA material from extracted mandibular first molar teeth to ensure the standardization of samples when evaluating antibacterial activity in the isthmus areas.

The differences observed among the experimental groups can be attributed to the temperature-dependent enhancement of NaOCI’s physicochemical and biological properties. Previous studies have shown that increasing the temperature of NaOCl significantly improves its ability to dissolve organic tissue, reduces surface tension, enhances diffusion, and increases its antibacterial activity [3,9]. In this study, it is likely that the 21°C and 45°C groups did not show significant differences due to the rapid equilibration of preheated NaOCl to body temperature upon contact with the canal walls [38]. However, the groups with intracanal heating (60°C and 150°C) likely maintained sufficient temperature during irrigation, allowing for more effective biofilm removal. The intracanal heat application provided by the heat carrier may have enabled improved penetration of NaOCl into the isthmus region and disrupted the biofilm matrix more efficiently. Therefore, based on the results obtained, the null hypothesis was rejected, and our findings confirmed that temperature plays a crucial role in the biofilm removal capacity of NaOCl.

The results of this study are consistent with several previous investigations that demonstrated the enhanced biofilm removal and antibacterial effectiveness of NaOCl when used at elevated temperatures. Sirtes et al. [3] reported that preheated NaOCl showed significantly improved tissue dissolution and antibacterial activity compared to room-temperature NaOCl. Iandolo et al. [8] further demonstrated that intracanal heating of NaOCl enhances its penetration and efficiency within anatomically complex areas, consistent with the superior outcomes observed at 60°C and 150°C in our study. Yared and Ramli [19] also confirmed that intracanal heating of NaOCl resulted in greater bacterial elimination compared to passive or sonic irrigation methods. Conversely, Pereira et al. [18] suggested that the chemical effects of heated NaOCl alone may not be sufficient when flow dynamics are limited, a finding that reinforces the importance of our controlled irrigation protocol. Taken together, our results validate and extend prior observations by confirming that temperature is a critical factor in enhancing NaOCl’s biofilm disruption capacity, particularly within the isthmus—a region notoriously difficult to clean using conventional irrigation techniques.

Various methods have been employed in the literature to evaluate root canal biofilms, including culture methods, molecular methods, fluorescence microscopy, confocal laser scanning microscopy (CLSM), and SEM. Molecular methods are considered highly sensitive for assessing bacterial load reduction. However, quantifying living and dying microorganisms together in these methods can yield misleading results [39]. Alves et al. reported similar results between PCR and culture methods when evaluating the remaining bacteria in the canal. CLSM is a widely used imaging method that provides direct, non-invasive visualization of biofilms with good spatial resolution [40].

Still, it cannot image the cellular superstructure and has limitations similar to light microscopy. SEM, on the other hand, offers a higher resolution than CLSM [41]. Studies frequently employ it to assess the effectiveness of different preparation and irrigation methods in removing debris, smear layer, and biofilm from root canals. SEM allows for examining the dentin surface at high magnifications, clearly visualizing biofilm presence [42]. In our study, SEM was used to observe the remaining biofilm structure in the isthmus area of artificial root canals, enabling visualization at high magnifications and resolutions. However, it should be noted that the processes involved in SEM imaging, such as fixation and dehydration, may lead to shrinkage of the biofilm structure, potentially affecting biofilm area calculations and serving as a limitation of this imaging technique.

A limitation of this study is that the biofilm model was based solely on *E. faecalis*, while clinical root canal infections typically involve complex polymicrobial communities. However, *E. faecalis* was selected due to its resistance and well-known association with persistent endodontic infections, as commonly reported in previous in vitro studies.

In our study, we employed a single biofilm model in an in vitro setting. Future studies can focus on evaluating the effectiveness of heated NaOCl on multi-type biofilm models to better simulate clinical conditions.

Our findings demonstrated that increasing the temperature of the NaOCl irrigation solution enhanced its ability to remove biofilm. Intracanal endodontic heat sources were identified as a more effective method for heating NaOCl. Heat activation of NaOCl was found to be particularly useful in achieving proper disinfection of isthmuses, which pose challenges for mechanical cleaning during endodontic treatment. The utilization of artificial root canals in our study allowed for standardization. However, it is worth noting that it may result in variations in biofilm adhesion to the surface compared to natural dentin bonding, a unique aspect of this research.

### Clinical significance

The results of this study provide important clinical insights into optimizing irrigation protocols in endodontic treatment. The enhanced effectiveness of NaOCl irrigation at elevated temperatures, particularly when heated intracanal, underscores the potential for improved disinfection in anatomically complex regions such as isthmuses. Since such areas are difficult to access with instruments alone, utilizing heat-activated NaOCl may help clinicians achieve more predictable bacterial elimination. This approach could be integrated into routine endodontic practice using existing heat carriers to enhance treatment outcomes without the need for additional or costly equipment.

## Conclusion

The results of our study demonstrate that heating NaOCl irrigation solutions within the root canal enhances biofilm removal, particularly from challenging anatomical areas such as isthmuses. Heat activation of NaOCl, especially when applied intracanal at 150°C, proved to be more effective than standard irrigation techniques at lower temperatures in removing *E. faecalis* biofilm. These findings suggest that incorporating intracanal-heated NaOCl into endodontic disinfection protocols may improve the clinical efficacy of root canal treatment by better addressing anatomically complex regions that are difficult to clean mechanically. Although the use of artificial root canal models allowed for high standardization, they may not fully replicate natural dentin properties; thus, differences in microbial adhesion and biofilm behavior should be considered. Further research involving polymicrobial biofilms, clinical applications are necessary to validate the in vivo effectiveness of thermally activated irrigation strategies.

## Supporting information

S1 Data Detailed raw measurements of total area, biofilm area (mm^2^), and biofilm percentage in the buccal, middle, and lingual isthmus regions across five experimental groups. This dataset includes sample-specific values that underpin the quantitative analysis presented in Fig 6.
(XLSX)

## References

[1] Chiniforush N, Pourhajibagher M, Shahabi S, Bahador A. Clinical approach of high technology techniques for control and elimination of endodontic microbiota. J Lasers Med Sci. 2015;6(4):139–50. doi: 10.15171/jlms.2015.09 26705458 PMC4688380

[2] Espir CG, Nascimento-Mendes CA, Guerreiro-Tanomaru JM, Cavenago BC, Hungaro Duarte MA, Tanomaru-Filho M. Shaping ability of rotary or reciprocating systems for oval root canal preparation: a micro-computed tomography study. Clin Oral Investig. 2018;22(9):3189–94. doi: 10.1007/s00784-018-2411-4 29525921

[3] Sirtes G, Waltimo T, Schaetzle M, Zehnder M. The effects of temperature on sodium hypochlorite short-term stability, pulp dissolution capacity, and antimicrobial efficacy. J Endod. 2005;31(9):669–71. doi: 10.1097/01.don.0000153846.62144.d2 16123703

[4] Dutner J, Mines P, Anderson A. Irrigation trends among American Association of Endodontists members: a web-based survey. J Endod. 2012;38(1):37–40. doi: 10.1016/j.joen.2011.08.013 22152617

[5] Jungbluth H, Marending M, De-Deus G, Sener B, Zehnder M. Stabilizing sodium hypochlorite at high pH: effects on soft tissue and dentin. J Endod. 2011;37(5):693–6. doi: 10.1016/j.joen.2011.02.019 21496673

[6] Spangberg L, Engström B, Langeland K. Biologic effects of dental materials: 3. Toxicity and antimicrobial effect of endodontic antiseptics in vitro. Oral Surgery, Oral Medicine, Oral Pathology. 1973;36(6):856–71. 4208832 10.1016/0030-4220(73)90338-1

[7] Tenore G, Palaia G, Ciolfi C, Mohsen M, Battisti A, Romeo U. Subcutaneous emphysema during root canal therapy: endodontic accident by sodium hypoclorite. Ann Stomatol (Roma). 2018;8(3):117–22. doi: 10.11138/ads/2017.8.3.117 29682224 PMC5897092

[8] Iandolo A, Amato M, Dagna A, Poggio C, Abdellatif D, Franco V, et al. Intracanal heating of sodium hypochlorite: Scanning electron microscope evaluation of root canal walls. J Conserv Dent. 2018;21(5):569–73. doi: 10.4103/JCD.JCD_245_18 30294123 PMC6161513

[9] Cunningham WT, Joseph SW. Effect of temperature on the bactericidal action of sodium hypochlorite endodontic irrigant. Oral Surg Oral Med Oral Pathol. 1980;50(6):569–71. doi: 10.1016/0030-4220(80)90443-0 6779248

[10] Gulabivala K, Aung TH, Alavi A, Ng YL. Root and canal morphology of Burmese mandibular molars. Int Endod J. 2001;34(5):359–70. doi: 10.1046/j.1365-2591.2001.00399.x 11482719

[11] De Deus QD. Frequency, location, and direction of the lateral, secondary, and accessory canals. J Endod. 1975;1(11):361–6. doi: 10.1016/s0099-2399(75)80211-1 10697487

[12] Capetillo J, Drum M, Reader A, Fowler S, Nusstein J, Beck M. Anesthetic efficacy of Intranasal 3% Tetracaine plus 0.05% Oxymetazoline (Kovanaze) in maxillary teeth. J Endod. 2019;45(3):257–62. doi: 10.1016/j.joen.2018.12.003 30803532

[13] Kayaoglu G, Ørstavik D. Virulence factors of Enterococcus faecalis: relationship to endodontic disease. Crit Rev Oral Biol Med. 2004;15(5):308–20. doi: 10.1177/154411130401500506 15470268

[14] Estrela C, Silva JA, de Alencar AHG, Leles CR, Decurcio DA. Efficacy of sodium hypochlorite and chlorhexidine against Enterococcus faecalis--a systematic review. J Appl Oral Sci. 2008;16(6):364–8. doi: 10.1590/s1678-77572008000600002 19082392 PMC4327704

[15] Murphy SV, Atala A. 3D bioprinting of tissues and organs. Nat Biotechnol. 2014;32(8):773–85. doi: 10.1038/nbt.2958 25093879

[16] Ordinola-Zapata R, Bramante CM, Duarte MAH, Cavenago BC, Jaramillo D, Versiani MA. Shaping ability of reciproc and TF adaptive systems in severely curved canals of rapid microCT-based prototyping molar replicas. J Appl Oral Sci. 2014;22(6):509–15. doi: 10.1590/1678-775720130705 24918662 PMC4307764

[17] Zou L, Shen Y, Li W, Haapasalo M. Penetration of sodium hypochlorite into dentin. J Endod. 2010;36(5):793–6. doi: 10.1016/j.joen.2010.02.005 20416421

[18] Pereira TC, Dijkstra RJB, Petridis X, Sharma PK, van de Meer WJ, van der Sluis LWM, et al. Chemical and mechanical influence of root canal irrigation on biofilm removal from lateral morphological features of simulated root canals, dentine discs and dentinal tubules. Int Endod J. 2021;54(1):112–29. doi: 10.1111/iej.13399 32880989 PMC7839520

[19] Yared G, Al Asmar Ramli G. Antibacterial ability of sodium hypochlorite heated in the canals of infected teeth: an ex vivo study. Cureus. 2020;12(2):e6975. doi: 10.7759/cureus.6975 32201655 PMC7075510

[20] Hsu YY, Kim S. The resected root surface. The issue of canal isthmuses. Dent Clin North Am. 1997;41(3):529–40. 9248689

[21] Qayyum S, Sharma D, Bisht D, Khan AU. Protein translation machinery holds a key for transition of planktonic cells to biofilm state in Enterococcus faecalis: A proteomic approach. Biochem Biophys Res Commun. 2016;474(4):652–9. doi: 10.1016/j.bbrc.2016.04.145 27144316

[22] Barut G, Haznedaroğlu F. Evaluation of the incidence and type of isthmus in mesial root canals of mandibular first and second molar teeth: a histological method. Yeditepe J Dent. 2016;12(1):23–7. doi: 10.5505/yeditepe.2016.32932

[23] Chávez de Paz LE, Hamilton IR, Svensäter G. Oral bacteria in biofilms exhibit slow reactivation from nutrient deprivation. Microbiology (Reading). 2008;154(Pt 7):1927–38. doi: 10.1099/mic.0.2008/016576-0 18599821

[24] Chávez de Paz LE, Bergenholtz G, Svensäter G. The effects of antimicrobials on endodontic biofilm bacteria. J Endod. 2010;36(1):70–7. doi: 10.1016/j.joen.2009.09.017 20003938

[25] Peters OA. Current challenges and concepts in the preparation of root canal systems: a review. J Endod. 2004;30(8):559–67. doi: 10.1097/01.don.0000129039.59003.9d 15273636

[26] Rôças IN, Siqueira JF Jr, Santos KRN. Association of Enterococcus faecalis with different forms of periradicular diseases. J Endod. 2004;30(5):315–20. doi: 10.1097/00004770-200405000-00004 15107642

[27] Stuart CH, Schwartz SA, Beeson TJ, Owatz CB. Enterococcus faecalis: its role in root canal treatment failure and current concepts in retreatment. J Endod. 2006;32(2):93–8. doi: 10.1016/j.joen.2005.10.049 16427453

[28] Torabinejad M, Fouad AF, Shabahang S. Endodontics E-Book. Elsevier Health Sciences. 2020.

[29] Moorer WR, Wesselink PR. Factors promoting the tissue dissolving capability of sodium hypochlorite. Int Endod J. 1982;15(4):187–96. doi: 10.1111/j.1365-2591.1982.tb01277.x 6964523

[30] Yildirim C, Karaarslan ES, Ozsevik S, Zer Y, Sari T, Usumez A. Antimicrobial efficiency of photodynamic therapy with different irradiation durations. Eur J Dent. 2013;7(4):469–73. doi: 10.4103/1305-7456.120677 24932123 PMC4053673

[31] Borzini L, Condò R, De Dominicis P, Casaglia A, Cerroni L. Root canal irrigation: chemical agents and plant extracts against Enterococcus faecalis. Open Dent J. 2016;10:692–703. doi: 10.2174/1874210601610010692 28217184 PMC5299586

[32] Leonardi DP, Grande NM, Tomazinho FSF, Marques-da-Silva B, Gonzaga CC, Baratto-Filho F, et al. Influence of activation mode and preheating on intracanal irrigant temperature. Aust Endod J. 2019;45(3):373–7. doi: 10.1111/aej.12336 30724420

[33] Teixeira FB, Sano CL, Gomes BPFA, Zaia AA, Ferraz CCR, Souza-Filho FJ. A preliminary in vitro study of the incidence and position of the root canal isthmus in maxillary and mandibular first molars. Int Endod J. 2003;36(4):276–80. doi: 10.1046/j.1365-2591.2003.00638.x 12702122

[34] von Arx T. Frequency and type of canal isthmuses in first molars detected by endoscopic inspection during periradicular surgery. Int Endod J. 2005;38(3):160–8. doi: 10.1111/j.1365-2591.2004.00915.x 15743419

[35] Swimberghe RCD, Coenye T, De Moor RJG, Meire MA. Biofilm model systems for root canal disinfection: a literature review. Int Endod J. 2019;52(5):604–28. doi: 10.1111/iej.13050 30488449

[36] Melchels FPW, Feijen J, Grijpma DW. A review on stereolithography and its applications in biomedical engineering. Biomaterials. 2010;31(24):6121–30. doi: 10.1016/j.biomaterials.2010.04.050 20478613

[37] Mohmmed SA, Vianna ME, Hilton ST, Boniface DR, Ng Y-L, Knowles JC. Investigation to test potential stereolithography materials for development of an in vitro root canal model. Microsc Res Tech. 2017;80(2):202–10. doi: 10.1002/jemt.22788 27813213

[38] Boutsioukis C, Psimma Z, van der Sluis LWM. Factors affecting irrigant extrusion during root canal irrigation: a systematic review. Int Endod J. 2013;46(7):599–618. doi: 10.1111/iej.12038 23289914

[39] Rôças IN, Lima KC, Siqueira JF Jr. Reduction in bacterial counts in infected root canals after rotary or hand nickel-titanium instrumentation--a clinical study. Int Endod J. 2013;46(7):681–7. doi: 10.1111/iej.12045 23331179

[40] Alves FRF, Rôças IN, Almeida BM, Neves MAS, Zoffoli J, Siqueira JF Jr. Quantitative molecular and culture analyses of bacterial elimination in oval-shaped root canals by a single-file instrumentation technique. Int Endod J. 2012;45(9):871–7. doi: 10.1111/j.1365-2591.2012.02045.x 22452547

[41] Bridier A, Dubois-Brissonnet F, Boubetra A, Thomas V, Briandet R. The biofilm architecture of sixty opportunistic pathogens deciphered using a high throughput CLSM method. J Microbiol Methods. 2010;82(1):64–70. doi: 10.1016/j.mimet.2010.04.006 20433880

[42] Teves A, Blanco D, Casaretto M, Torres J, Alvarado D, Jaramillo DE. Effectiveness of different disinfection techniques of the root canal in the elimination of a multi-species biofilm. J Clin Experim Dentist. 2019;11(11):e978. 10.4317/jced.56000 PMC6825738 31700570

